# Time-course microarrays reveal early activation of the immune transcriptome and adipokine dysregulation leads to fibrosis in visceral adipose depots during diet-induced obesity

**DOI:** 10.1186/1471-2164-13-450

**Published:** 2012-09-04

**Authors:** Eun-Young Kwon, Su-Kyung Shin, Yun-Young Cho, Un Ju Jung, Eunjung Kim, Taesun Park, Jung Han Yoon Park, Jong Won Yun, Robin A McGregor, Yong Bok Park, Myung-Sook Choi

**Affiliations:** 1 Center for Food and Nutritional Genomics Research, Kyungpook National University, Daegu, Republic of Korea; 2 Department of Food Science and Nutrition, Catholic University of Daegu, Gyeongsan, Republic of Korea; 3 Department of Food and Nutrition, Yonsei University, Seoul, Republic of Korea; 4 Department of Food Science and Nutrition, College of Natural Sciences, Hallym University, Chuncheon, Republic of Korea; 5 Department of Biotechnology, Daegu University, Kyungsan, Kyungbuk, Republic of Korea; 6 School of Life Science & Biotechnology, Kyungpook National University, Daegu, Republic of Korea; 7 Department of Food Science and Nutrition, Kyungpook National University, 1370 Sank-Yuk Dong Puk-Ku, Daegu, 702-701, Korea

**Keywords:** Adipocytokine dysregulation, Transcriptional response, Adipose tissue, Extracellular matrix, Cathepsin, Fibrosis

## Abstract

**Background:**

Visceral white adipose tissue (WAT) hypertrophy, adipokine production, inflammation and fibrosis are strongly associated with obesity, but the time-course of these changes *in-vivo* are not fully understood. Therefore, the aim of this study was to establish the time-course of changes in adipocyte morphology, adipokines and the global transcriptional landscape in visceral WAT during the development of diet-induced obesity.

**Results:**

C57BL/6 J mice were fed a high-fat diet (HFD) or normal diet (ND) and sacrificed at 8 time-points over 24 weeks. Excessive fat accumulation was evident in visceral WAT depots (Epidydimal, Perirenal, Retroperitoneum, Mesentery) after 2–4 weeks. Fibrillar collagen accumulation was evident in epidydimal adipocytes at 24 weeks. Plasma adipokines, leptin, resistin and adipsin, increased early and time-dependently, while adiponectin decreased late after 20 weeks. Only plasma leptin and adiponectin levels were associated with their respective mRNA levels in visceral WAT. Time-course microarrays revealed early and sustained activation of the immune transcriptome in epididymal and mesenteric depots. Up-regulated inflammatory genes included pro-inflammatory cytokines, chemokines (Tnf, Il1rn, Saa3, Emr1, Adam8, Itgam, Ccl2, 3, 4, 6, 7 and 9) and their upstream signalling pathway genes (multiple Toll-like receptors, Irf5 and Cd14). Early changes also occurred in fibrosis, extracellular matrix, collagen and cathepsin related-genes, but histological fibrosis was only visible in the later stages.

**Conclusions:**

In diet-induced obesity, early activation of TLR-mediated inflammatory signalling cascades by CD antigen genes, leads to increased expression of pro-inflammatory cytokines and chemokines, resulting in chronic low-grade inflammation. Early changes in collagen genes may trigger the accumulation of ECM components, promoting fibrosis in the later stages of diet-induced obesity. New therapeutic approaches targeting visceral adipose tissue genes altered early by HFD feeding may help ameliorate the deleterious effects of diet-induced obesity.

## Background

White adipose tissue (WAT) not only serves as a fuel storage depot but also releases a variety of bioactive factors, called adipokines, which have pro-and anti-inflammatory activity. In obesity, most adipokines, including leptin, resistin and tumor necrosis factor alpha (TNFα), are overproduced and promote inflammation. Whereas, the production of anti-inflammatory adipokines, such as adiponectin, is decreased with increasing adiposity. Since, inflammation is considered to be causal in the development of obesity and insulin resistance [1-3], the dysregulated production of adipokines caused by excessive adipose tissue expansion is linked to the pathophysiology of obesity-related metabolic syndrome [4].

There is a close relationship between inflammation and the extracellular matrix (ECM), which requires remodelling to accommodate adipocyte growth during adipose tissue expansion. In obesity, adipose tissue inflammation is further exacerbated by excessive accumulation of ECM components in adipose tissue, possibly as a result of pro-inflammatory cytokines released by adipocytes [5]. Excessive accumulation of ECM components may represent a key event in obesity-mediated adipose tissue fibrosis, similar to fibrosis in other tissues such as liver [6,7]. Acumulating evidence indicates that altered circulating adipokine levels contribute to the progression of cardiac or hepatic fibrosis [8,9]. Thus, adipokine dysregulation may also regulate remodeling of the adipocyte ECM and fibrosis. However, the actual time-course of adipokine dysregulation, inflammation, ECM accumulation leading to the development of adipose tissue fibrosis is yet to be fully established.

High-throughput microarrays assessing global gene expression changes provide a unique window into the molecular events occurring in adipose tissue during the development of obesity. Previous microarray studies have revealed that a high fat diet (HFD) is associated with adipokine dysregulation, inflammation and fibrosis [10-15]. However, these microarray studies tend to compare gene expression differences at a single end-point only or over short-time periods between 2–12 weeks. Following the molecular changes in adipose tissue during the development of obesity in humans is not feasible. Therefore, time-course models of diet-induced obesity in mice can help improve our understanding of the molecular pathogenesis of obesity *in-vivo*. However, the use of genetically obese animals or extremely high levels of dietary fat intake, which do not reflect normal dietary consumption hampers the translation of findings from mouse obesity models to humans [10-14]. Therefore we established a long-term diet-induced obesity model, by feeding C57BL/6 J mice with a high-fat diet (39%kcal) or normal diet for 24 weeks and sacrificing mice at regular time-points to allow in-depth time-course microarray analysis of visceral adipose tissue depots.

Accordingly the aim of the present study was to establish the time-course of changes in adipocyte morphology, adipokines and the global transcriptional landscape in visceral adipose tissue depots during the transition from a lean to obese phenotype in HFD fed mice. We primarily focus on the timing of changes associated with adipokine levels, inflammation and fibrosis, which are recognized as important factors associated with human obesity.

## Methods

### Animals

Three hundred and sixty male inbred C57BL/6 J mice were obtained from the Jackson Laboratory (Bar Harbor, ME) at 4 weeks of age. All the mice were individually housed under a constant temperature (24°C) and 12-hour light/dark cycle, fed the AIN-76 semi-purified diet for one week acclimation period after arrival, and then randomly divided into a normal diet (ND) and an HFD group, with 18 mice per group for 0, 2, 4, 6, 8, 12, 16, 20 and 24 weeks. After the acclimation, the HFD group was fed the AIN-76 semi-purified diet with 39.2 kcal% fat (American Institute of Nutrition, 1977) consisting of 20% fat and 1% cholesterol ( Additional file 1: Table S1). Every 2–4 weeks, after a 12 h fast, blood samples were drawn from the inferior vena cava. Blood was collected in heparin-coated tubes, centrifuged at 1,000 × g for 15 min at 4°C and then stored at −70°C. Mice were anaesthetized and sacrificed at 0, 2, 4, 6, 8, 12, 16, 20 and 24 weeks. The adipose tissues were then removed, rinsed, weighed, immediately frozen in liquid nitrogen, and stored at −70°C until further analysis. The current study protocol was approved by the Ethics Committee for animal studies at Kyungpook National University, Republic of Korea.

### Plasma adipokines

Radioimmunometric assays were performed for the measurement of plasma amylin (amylin RIA kit; Diagnostic Systems Laboratories), adipsin (adipsin RIA kit; PerkinElmer Life and Analytical Sciences, Boston, MA), adiponectin (adiponectin RIA kit; PerkinElmer Life and Analytical Sciences, Boston, MA), leptin (Mouse leptin RIA kit; Linco Research, St. Charles, MO), and resistin concentrations (resistin RIA kit; PerkinElmer Life and Analytical Sciences, Boston, MA).

### Morphology of fat tissues

Epididymal adipose tissue was fixed in 10% (v/v) paraformaldehyde/PBS and embedded in paraffin for staining with hematoxylin and eosin (H&E) and Masson’s trichrome. The stained area was viewed using a microscope at a magnification of 200× and 400×, respectively.

### RNA preparation and quality control

Total RNA was extracted from the adipose tissue using TRIZOL reagent (Invitrogen Life Technologies, Grand Island, NY) according to the manufacturer’s instructions. DNase digestion was used to remove any DNA contamination and RNA was re-precipitated in ethanol to ensure no phenol contamination. For quality control, RNA purity and integrity were evaluated using the Agilent 2100 Bioanalyzer (Agilent Technologies, Palo Alto, USA). For epididymal adipose tissue, six pooled RNA sample sets were constructed for the ND and HFD groups at each time-point as described previously [16]. In mesenteric adipose tissue, one pooled RNA sample set was constructed for the ND and HFD groups at each time-point based on 18 individual samples. RNA was stored at −70°C prior to further analysis by microarray and RT-qPCR.

### Microarray analysis

Total RNA was amplified and purified using the Ambion Illumina RNA amplification kit (Ambion, Austin, USA) to yield biotinylated cRNA according to the manufacturer’s instructions. After purification, the cRNA was quantified using the ND-1000 Spectrophotometer (NanoDrop, Wilmington, USA). 750 ng of labelled cRNA samples were hybridized to each Illumina MouseWG-6 v2 Expression BeadChip for 16–18 h at 58°C, according to the manufacturer's instructions (Illumina, Inc., San Diego, USA). Detection of array signal was carried out using Amersham fluorolink streptavidin-Cy3 (GE Healthcare Bio-Sciences, Little Chalfont, UK) following the bead array manual. The quality of hybridization and overall chip performance were monitored by visual inspection of both internal quality controls and the raw scanned data. Raw data was extracted using the Illumina BeadStudio software provided by the manufacturer. Probe signal intensities significantly higher than background intensities were determined (detection p-value > 0.05), but probe signal data were not filtered to preserve probes with low expression at different time points. Probe signal intensities were quantile normalized and log transformed. Microarray analysis was performed in ArrayAssist (Stratagene, USA), Bioconductor and R programing language. LIMMA was used to determine significant differentially expressed genes (HFD responsive genes) between HFD- and ND-fed mice at each time point based on FDR < 5%, Benjamin and Hochberg adjusted *p*-value <0.05 and log fold change >1 [17]. This microarray data was deposited in Gene Expression Omnibus (GEO) database (GEO accession numbers: GSE39549).

### RT-qPCR

The same total RNA (1 μg) samples that were hybridised to microarrays were reverse-transcribed into cDNA using the QuantiTect® reverse transcription kit (Qiagen, Germany). Then mRNA expression was quantified by real-time quantitative PCR, using the SYBR green PCR kit (Qiagen, Germany) and the CFX96^TM^ real-time system (BIO-RAD). Gene specific mouse primers were used to detect leptin, adiponectin and resistin (Additional file 2: Table S2). Cycle thresholds were determined based on SYBR green emission intensity during the exponential phase. Ct data were normalized using Gapdh, which was stably expressed in both HFD and ND fed mice. Relative gene expression was calculated using the 2^−△△^Ct method [18].

### Functional features of high-fat feeding responsive genes in visceral adipose tissues

To identify which biological processes were associated with the visceral adipose tissue genes altered by HFD at one or more time-points over 24 weeks, enriched functional features were identified using DAVID [19]. The analysis was performed using Gene Ontology and Pathways and mapped by DAVID ID. Then the DAVID Functional Annotation Clustering tool [19] was applied to identify enriched biological themes and cluster redundant annotation terms.

### Identification of genes that respond to both age and high-fat diet simultaneously in epididymal adipose tissue

Next, we examined which HFD-responsive genes in epididymal adipose tissue were correlated with age and/or body mass. At first, genes affected by advancing age were identified by examining genes that were continuously up- or down-regulated in the both ND and HFD group. These genes were overlaid with the HFD-responsive genes (HFD vs. ND, fold change > 2 and p-value < 0.05) to screen genes that responded to both age and high-fat diet simultaneously in epididymal adipose tissue. These overlapped genes were selected and correlated with body mass.

### Statistical analysis

The parameter values were expressed as the mean (standard error of the mean (SEM). Significant differences between groups were determined by student’s t-test and wilcoxon t-test using the SPSS program (SPSS Inc., Chicago, IL). Correlation analyses utilized the Pearson’s coefficient. Results were considered statistically significant at p < 0.05.

## Results

### Time-course of changes in body weight and food efficiency during diet-induced obesity

Significant increases of body weight gain and energy consumption were observed in the HFD group after the 4^th^ week and 2^th^ week, respectively. The food efficiency ratio (FER) was also significantly increased in the HFD group after the 4^th^ week of high-fat feeding (Additional file 3:Table S3) [20] compared to the ND group.

### Time-course of changes in WAT depot weights, adipocyte size and adipose tissue fibrosis during diet-induced obesity

**Figure 1:**
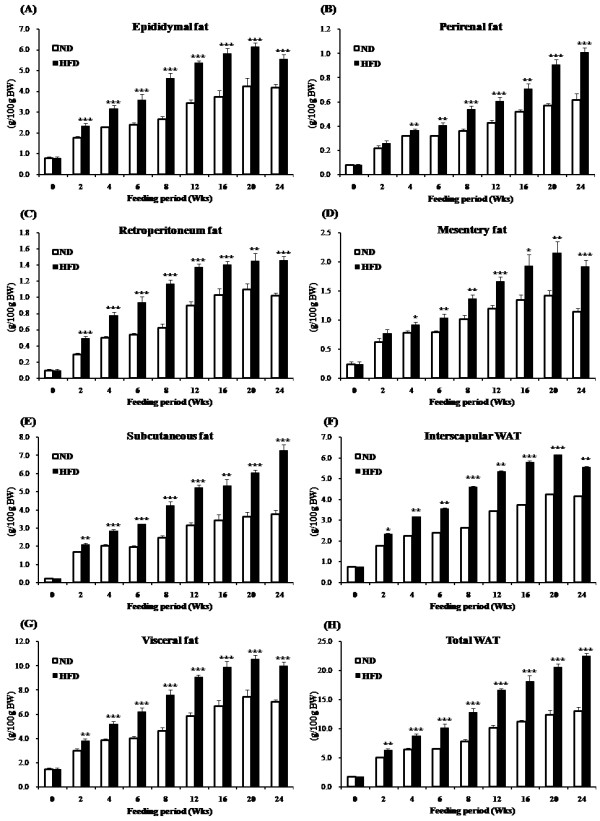
**Timely effect of high-fat feeding on adipose tissue weights over 24 weeks in C57BL/6 J mice.** Data shown as means ± S.D. Values are significantly different from the ND group according to Student's test: *p < 0.05, **p < 0.01, ***p < 0.001. ND: normal diet (AIN-76), HFD: high-fat diet (20% fat, 1% cholesterol), WAT: white adipose tissue.

**Figure 2:**
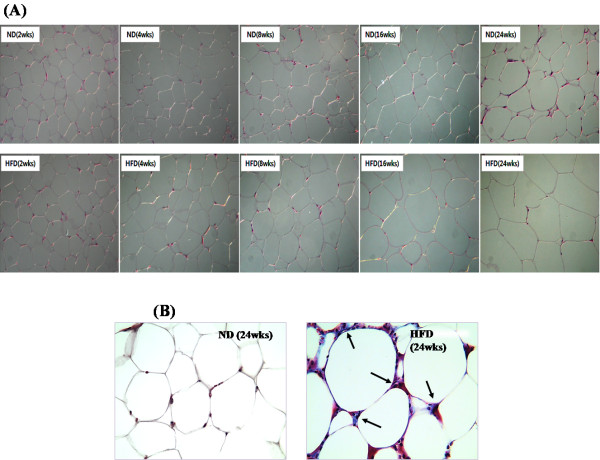
**Timely effect of high-fat feeding over 24 weeks on (A) H&E and (B) Masson’s trichrome staining of epididymal white adipose tissue in C57BL/6 J mice.** (**A**) H&E stained transverse-section of epidydimal fat. Original magnification × 200. (**B**) Masson’s trichrome stained transverse-section of epidydimal fat. Fibrillar collagens, primarily collagen I and III, are stained with blue as indicated with arrowheads. Nuclei and keratin are stained with deep purple and red, respectively. Original magnification × 400. ND: normal diet (AIN-76), HFD: high-fat diet (20% fat, 1% cholesterol).

When WAT weights were expressed in g/100 g WAT, visceral WAT depots including epididymal, perirenal, retroperitoneum, mesentery WAT were significantly increased from week 2 in the HFD group compared to ND group. Similarly, from week 2, subcutaneous WAT was significantly higher in the HFD group (Figure 1). In addition to increased body fat, adipocyte size was also enlarged in the epididymal WAT depot in a time-dependent manner (Figure 2A).

To assess fibrosis Masson's trichrome staining was performed, which revealed the epididymal WAT depot of the HFD group contained very pronounced trichrome-positive “streaks” interspersed among the adipocytes at 24 weeks. Whereas, the epididymal WAT depot of the ND group revealed a normal fat-pad, with densely packed and very thin collagen sheets surrounding each adipocyte at week 24 (Figure 2B).

### Time-course of changes in plasma adipokine, glucose, insulin and lipids levels during diet-induced obesity

**Figure 3:**
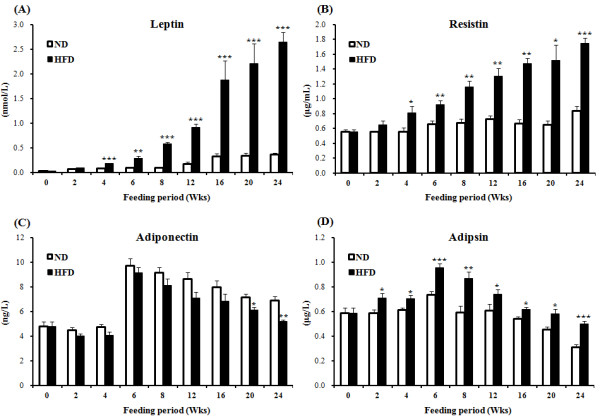
**Time-resolving effect of high-fat feeding over 24 weeks on plasma concentration of (A) leptin, (B) resistin, (C) adiponectin and (D) adipsin in C57BL/6 J mice.** Data shown as means ± S.D. Values are significantly different from the ND group according to Student's test: *p < 0.05, **p < 0.01, ***p < 0.001. ND: normal diet (AIN-76), HFD: high-fat diet (20% fat, 1% cholesterol).

The time-course of changes in plasma adipokine levels in response to HFD is shown in Figure 3. The most prominent changes were observed in leptin and resistin levels, which were increased from week 4 to week 24 in the HFD group. Plasma adipsin levels were also elevated in the HFD group from week 4, reaching a peak at week 6 and thereafter gradually decreasing. In contrast, plasma adiponectin levels in the HFD group tended to be lower than in the ND group for the overall feeding period and were significantly lower at week 20 and 24. Fasting blood glucose and insulin resistance and plasma total cholesterol concentration were markedly elevated in the HFD group after 16 week and 6 weeks, respectively. No differences in plasma TG and FFA levels were detectable between the HFD group and ND group at any time points (data not shown) [21].

### Time-course of transcriptional changes in epididymal and mesenteric WAT depots during diet-induced obesity

In the epididymal WAT depot, 2037 genes were altered at one or more time points in response to HFD intake over 24 weeks (Table 1), of which 1610 genes were up-regulated and 445 genes were down-regulated. In mesenteric WAT depot, 1784 genes were altered at one or more time point in response to HFD intake over 24 weeks, of which 1134 genes were up-regulated and 892 genes were down-regulated (Table 1). Functional gene ontology terms associated with these HFD responsive genes in the epididymal WAT and mesenteric WAT depots were clustered using DAVID (Table 2). Immune and inflammatory system function terms were enriched among the consistently up-regulated genes across all time-points, in response to HFD, in both the epididymal and mesenteric WAT depots (Table 2A). Glucose metabolic system function terms were enriched among the down-regulated genes (Table 2B). Lipid and cholesterol metabolism related functional gene ontology terms were specifically enriched among the down-regulated genes in the mesenteric WAT depot across all time-points.

**Table 1:** Number of high-fat diet responsive genes in two visceral WAT depots at multiple time-points in C67BL/6 J mice

	**Epididymal WAT**			**Mesenteric WAT**		
**Time (week)**	**Fold-changed genes**	▴	▼	**Fold-changed genes**	▴	▼
**2**	1478	1233	245	999	684	315
**4**	3	1	2	190	177	13
**8**	37	34	3	306	49	257
**20**	339	200	139	247	32	215
**24**	431	341	90	751	445	306
**All**	2037	1610	445	1784	1134	892

Differentially expressed genes based on HFD v ND comparison at each time-point according to p-value < 0.05, fold change > 2 in the epididymal WAT depot and fold change > 2 in the mesenteric WAT depot. Unique HFD responsive genes were differentially expressed at one or more time-points.

**▴:** up-regulated, **▼:** down-regulated, WAT: white adipose tissue.

**Table 2:** Functional annotation gene clusters (A) up-regulated and (B) down-regulated in visceral adipose tissue depots by high-fat feeding in C57BL/6 J mice over 24 weeks

	**Depot**	**E.S.**	**Functional terms**	**No. of genes**	**p-value**
(A)	Epididymal WAT	5.34	Inflammatory response	41	1.10E-06
			Response to wounding	53	6.40E-06
			Defense response	62	8.20E-06
		2.59	Regulation of phagocytosis	10	4.20E-05
		2.23	Cell activation	38	5.60E-05
		2.09	Positive regulation of immune system process	37	2.30E-06
			Positive regulation of immune response	25	8.40E-05
	Mesenteric WAT	4.84	Positive regulation of immune system process	32	3.80E-08
			Positive regulation of immune response	25	5.40E-08
			Activation of immune response	17	4.40E-06
		3.48	Defense response	47	3.40E-06
		2.72	Cell activation	32	2.10E-06
			Leukocyte activation	28	1.50E-05
			Lymphocyte activation	25	3.10E-05
(B)	Epididymal WAT	5.77	Fatty acid metabolic process	20	1.60E-08
		3.55	Hexose metabolic process	20	2.80E-09
			Monosaccharide metabolic process	21	3.40E-09
			Glucose metabolic process	17	3.40E-08
	Mesenteric WAT	12.62	Glucose metabolic process	36	2.80E-18
			Monosaccharide metabolic process	40	8.20E-17
			Hexose metabolic process	37	4.80E-16
			Glycolysis	17	7.60E-12
			Gluconeogenesis	7	9.10E-05
		4.41	Steroid metabolic process	26	3.80E-08
			Sterol metabolic process	16	9.10E-07
			Cholesterol metabolic process	15	1.50E-06
			Lipid biosynthetic process	32	1.90E-06
			Lipid catabolic process	20	5.60E-06
			Acetyl-CoA metabolic process	9	3.70E-05

Functional annotation terms enriched amongst the up/down regulated genes in epididymal and mesenteric WAT of HFD fed C57BL/6 J mice over 24 weeks. Functional annotation terms were clustered according to biological processes. WAT: white adipose tissue; E.S.: Enrichment Score.

### Epididymal WAT depot genes altered by advancing age and diet-induced obesity

**Figure 4:**
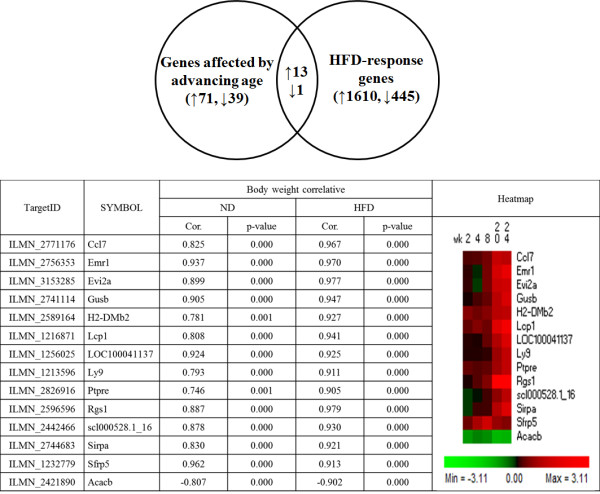
**The list of high-fat diet responding gene transcripts in epididymal WAT that simultaneously associated with advancing age in ND and HFD groups for 24 weeks.** The gene list affected by advancing age was based on continuously up- or down-regulated genes in both ND and HFD group. HFD-responsive genes were based on HFD vs ND, fold change > 2 and p-value < 0.05. ND: normal diet (AIN-76), HFD: high-fat diet (20% fat, 1% cholesterol), Cor.: Correlation Coefficient.

When HFD-responsive genes, in epididymal WAT, were overlapped with age-dependent genes, 14 genes were identified as shown in Figure 4. Fold changes and q-values of identified 14 genes are given in Additional file 4: Table S4. These 14 genes were correlated with body weight in both ND and HFD groups. Accordingly, these genes were associated with not only with age but also diet-induced obesity. In particular, among these 14 age and HFD responsive genes was EGF-like module containing, mucin-like, hormone receptor-like sequence 1 (*Emr1*) and chemokine (C-C motif) ligand 7 (*Ccl7*), which are both genes associated with inflammation, as well as body weight gain.

### Time-course of transcriptional changes in adipokine, pro-inflammatory cytokine, chemokine and upstream signalling genes in visceral WAT depots during diet-induced obesity

**Figure 5:**
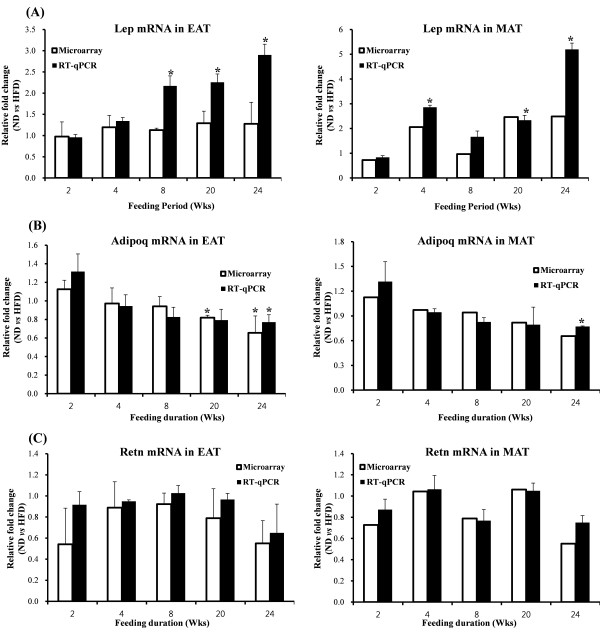
**RT-qPCR validation.** Time-dependent fold changes in (**A**) Lep mRNA, (**B**) Adipoq mRNA and (**C**) Retn mRNA expression based on HFD vs.ND group over 24 weeks. Microarray and RT-qPCR data shown as means ± S.D. *p < 0.05 based on wilcoxon t-test and student’s t-test, respectively. No statistical analysis, since we evaluated 1 pooled sample for microarray analysis in MAT. ND: normal diet (AIN-76), HFD: high-fat diet (20% fat, 1% cholesterol), EAT: epididymal adipose tissue, MAT: mesenteric adipose tissue, Lep: leptin, Adipoq: adiponectin, Retn: resistin.

The time-course of pro- and anti-inflammatory adipokine gene expression changes in WAT is shown in Figure 5. Consistent with plasma leptin and adiponectin levels, leptin mRNA expression was also up-regulated, while adiponectin mRNA expression was down-regulated consistently during the development of diet-induced obesity over 24 weeks. Microarray validation using via RT-qPCR revealed agreement in the time-course and pattern of changes in gene expression. There was no significant change in resistin mRNA expression.

**Figure 6:**
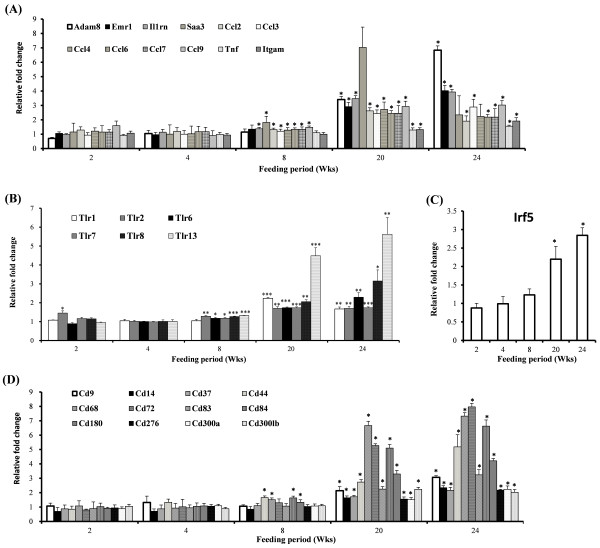
**Effect of high-fat feeding on transcription of (A) pro-inflammatory cytokine and chemokine genes, (B) Toll-like receptors, (C) IRF5 and (D) CD families expressed in epididymal adipose tissue of C57BL/6 J mice over 24 weeks (HFD vs. ND group).** mRNA expression in epididymal adipose tissue measured by the microarray analysis. Fold changes in mRNA expression (HFD vs. ND) were displayed at each time point over 24 weeks. Data shown as means ± S.D. *p < 0.05 based on wilcoxon t-test. ND: normal diet. HFD: high-fat diet.

During the development of diet-induced obesity, transcription of several pro-inflammatory cytokines and chemokines such as disintegrin and metallopeptidase domain 8 (*Adam8*), *Emr1*, interleukin 1 receptor antagonist (*Il1rn*), tumor necrosis factor (*Tnf*), serum amyloid A 3 (*Saa3*), integrin alpha-M (*Itgam*), chemokine (C-C motif) ligand 2 (*Ccl2*), ligand 3 (*Ccl3*), ligand 4 (*Ccl4*), ligand 6 (*Ccl6*), ligand 7 (*Ccl7*) and ligand 9 (*Ccl9*), were significantly elevated in epididymal WAT between week 20 and 24 (Fig. 6A). Moreover, gene expression of toll-like receptors (TLRs), interferon regulatory factor 5 (*Irf5*) and CD antigen families were also significantly altered by HFD in epididymal WAT. Among them *Tlr2*, *Tlr6*, *Tlr7*, *Tlr8*, *Tlr13* and *Trl1*, *Irf5* were significantly up-regulated by HFD from week 8 and week 20, respectively (Figure 6B, C). Expression of *Cd9, Cd14, Cd37, Cd44, Cd68, Cd72, Cd83, Cd84, Cd180, Cd276, Cd300a* and *Cd300lb* was also significantly elevated from week 8 to 24 in a time dependent manner (Figure 6D).

### Time-course of transcriptional changes in extracellular matrix genes, adipose tissue-expressed collagen genes and cathepsin family genes during diet-induced obesity

**Figure 7:**
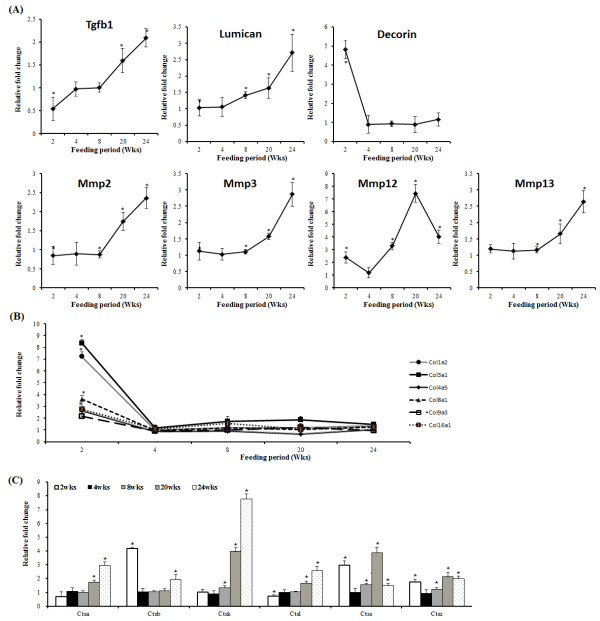
**Effect of high-fat feeding on transcription of key fibrotic genes in epididymal adipose tissue of C57BL/6 J mice over 24 weeks (HFD vs. ND group).** (**A**) Extracellular matrix genes (TGF-β1, decorin, lumican and several MMPs), (**B**) adipose tissue-expressed collagen genes and (**C**) cathepsin genes in the adipose tissue measured by time-course microarrays. Fold changes in mRNA expression (HFD vs. ND) were displayed at each time-point over 24 weeks. Data shown as means ± S.D. *p < 0.05 based on wilcoxon t-test. ND: normal diet. HFD: high-fat diet.

To identify the time-dependent effect of HFD on fibrosis relative to the ND group, we examined the levels of many key fibrotic genes (Figure 7). Lumican (*Lum*) and transforming growth factor β (*Tgfb1*) mRNA levels were up-regulated, while decorin (*Dcn*) mRNA levels were down-regulated by HFD in a time dependent manner. Several matrix metalloproteases (*Mmp)* showed time-dependent transcriptional alterations in response to HFD. *Mmp*2, *Mmp*3 and *Mmp*13 mRNA levels were up-regulated by HFD from week 8 to 24, whereas *Mmp12* mRNA levels were significantly up-regulated earlier after week 2 in the HFD group (Figure 7A). Collagen-related mRNA levels including *Col1a2, Col3a1, Col4a5, Col8a1, Col9a3* and *Col16a1* were significantly higher at week 2 in the HFD group, relative to the ND group, and sharply decrease after 4 weeks (Figure 7B). Cathepsin (*Cts*) mRNA levels were also altered by HFD (Figure 7C), *Ctsa, Ctsb, Ctsk, Ctsl, Ctss* and *Ctsz* mRNA levels were mostly up-regulated by high-fat feeding in the epididymal WAT depot. In particular, *Ctsk* mRNA levels were significantly increased from week 8 in the HFD group. *Ctss* and *Ctsz* mRNA levels were significantly higher at week 2, 8, 20 and 24, while *Ctsb* mRNA levels were only higher at week 2 and week 24 in the HFD group.

## Discussion

Adipose tissue can rapidly expand in response to excess energy intake through both adipocyte hypertrophy (cell size increase) and hyperplasia (cell number increase). Excessive adipose tissue expansion in obesity is associated with adipokine dysregulation, inflammation, ECM remodeling and fibrosis particulary in visceral adipose depots. However, the time-course of these pathophysiological events and the underlying transcriptional changes in adipose tissue are not fully understood. We show adipose tissue expansion occurs in multiple visceral WAT depots early during the development of diet-induced obesity. Pro-inflammatory adipokine levels also increase early after 2–4 weeks, while anti-inflammatory adipokine levels are reduced after 20 weeks in HFD fed mice. Time-course microarray profiling revealed early and prolonged up-regulation of inflammation and immune system associated genes in the epididymal WAT depot and the smaller mesenteric WAT depot. In the later stages of diet-induced obesity, fibrosis was evident in the epididymal WAT depot. Hence the long-term diet-induced obesity model used in the present study provides a unique insight into the timing of events in visceral adipose tissue during the development of obesity.

Adipocytokine dysregulation is reported to be closely associated with obesity and plasma proinflammatory adipokines are widely reported to be elevated in obese animals. Adipokines are secreted from visceral adipose depots, hence expansion of visceral adipose tissue depots is correlated with plasma adipokine levels, but it is less well established whether adipokine are transcriptionally regulated in obesity. In the present study, plasma leptin and resistin levels were increased consistently from 4 to 24 weeks. Time-course transcriptional profiling revealed leptin mRNA levels were increased in both the epididymal and mesenteric WAT depots over 24 weeks, but changes in resistin mRNA levels were less clear. Notably, leptin mRNA levels were higher in the mesenteric WAT depot than in the epididymal WAT depot. From a structural perspective, leptin is a member of the cytokine family, and leptin receptors belong to the class-I cytokine receptor family [22,23]. Leptin can control the production and activation of pro-inflammatory cytokines such as IL-6 and TNF-α by macrophages [24,25], and is a key regulatory factor expressed in both zebrafish and mammalian obesity in lipid metabolism [26]. Resistin is also known as an adipose tissue-specific secretory factor, participating in the pathogenesis of insulin resistance, adipogenesis and inflammation in mice [27,28]. However, the time-course of plasma resistin level changes, was largely dissociated from resistin mRNA levels in the epididymal and mesenteric WAT depots. Jackson et al. [29] reported similar observations, largely that resistin mRNA levels were very low or undetectable in adipose tissue, which suggests that visceral adipocytes are not a major cell type producing resistin. Plasma resistin levels may by due to resistin production by circulating monocytes and macrophages. Like many other pro-inflammatory cytokines, resistin stimulates intracellular signalling through NF-κB activation, which in turn promotes the synthesis of other pro-inflammatory cytokines, including MCP-1, vascular cell adhesion molecule-1 (VCAM-1), as well as intercellular adhesion molecule-1 (ICAM-1), which are involved in chemotactic pathways directing leukocyte recruitment to sites of infection [30,31]. Therefore, while resistin appears not to be produced by visceral adipose tissue depots, plasma resistin may still be a player in early visceral adipose tissue inflammation via activation of pro-inflammatory signalling and leukocyte recruitment.

Circulating plasma adiponectin and adiponectin mRNA levels are reportedly decreased by HFD and inversely correlated with fat mass, and directly correlated with insulin sensitivity [32,33]. In the present study, a markedly different time-course in plasma adiponectin levels was evident with late decreases in plasma adiponectin observed, along with concomitant down-regulation of adiponectin mRNA levels in both the epididymal and mesenteric WAT depots. There have been reports indicating adiponectin is probably not a useful early biomarker of obesity. Sumiyoshi and colleagues reported that high fat feeding (45% fat, wt/wt) for 55 weeks did not alter circulating adiponectin level in C57BL/6 J mice compared with a low fat diet (3% fat, wt/wt) [34]. In accordance with our results, Park and colleague’s reported that circulating adiponectin level was significantly reduced after 20 weeks of high-fat feeding (55 kcal% from fat) compared with a normal diet [35]. The lack of any early changes in plasma adiponectin and visceral adipose tissue adiponectin mRNA levels in HFD fed mice may be part of an adaptive response to protect against early inflammatory changes. There were prominent changes in adiponectin levels in both HFD and ND fed mice around 6 weeks, which we speculate may be related to sexual maturation or ageing.

Adipsin, a serine protease is synthesized and secreted by adipose cells and is found in the bloodstream [36]. In the current study, plasma adipsin levels were significantly higher in the HFD group compared to ND group from the week 2 to 24. However, it is not clear whether the higher concentration of adipsin in blood reflects increased adiposity in the HFD fed mice, because fat mass was increased over time but plasma adipsin decreased after 6 weeks regardless of diet. Previous studies have reported that blood adipsin levels are substantially increased in obese humans [37,38], but decreased in murine models of obesity [39]. Taken together, the present *in-vivo* observations of the time-course of various plasma adipokine levels and visceral WAT depot adipokine mRNA levels suggests leptin gene expression is up-regulated early, while adiponectin gene expression is down-regulated later in the epididymal and mesenteric WAT depots contributing to time-dependent changes in plasma adipokine levels.

We conducted in-depth time-course microarray profiling. The early and prolonged changes in the global transcriptome of both epididymal and mesenteric WAT revealed up-regulation of genes predominantly associated with immune and inflammatory system function enriched across all time-points in HFD fed mice. On the other hand, down-regulated genes were predominantly associated with monosaccharide, hexose and glucose metabolism across all time-points. Interestingly, down-regulated genes were also associated with lipid and cholesterol metabolism across all time-points although predominantly in the mesenteric WAT depot, rather than the epididymal WAT depot. In agreement with other diet-induced obesity models, functional ontology terms including sterol metabolism and acetyl-CoA biosynthesis were reportedly highly enriched among regulated genes in the mesenteric WAT depot but not in the epididymal WAT depot [40], and transcripts involved in lipid biosynthesis were up-regulated only by low fat meals [33]. Genes associated with the inflammatory response that were significantly up-regulated in the epididymal WAT depot during diet-induced obesity included pro-inflammatory cytokine and chemokine genes (*TnfIl1rnSaa3Emr1Adam8ItgamCcl2Ccl*3, *Ccl*4, *Ccl*6, *Ccl*7 and *Ccl*9) and their upstream signalling pathway genes such as TLRs, *Irf5* and *Cd14*. In accordance with our study, many inflammatory genes including Ccl3, Ccl2, Saa3, Tlr13, which were highly expressed in adipose tissue of fat mouse, have been linked with obesity and/or insulin resistance [41], furthermore *Emr1* and *Ccl7* mRNA expression are associated with body weight gain as well as obesity-related inflammation. Adipose tissue consists of multiple cell types including adipocytes, fibroblasts, macrophages and endothelial cells. Therefore, the genes identified in the present study as differentially expressed during the development of diet-induced induced obesity will reflect overall changes in visceral adipose tissue depots rather than specific cell-types.

Recent studies have suggested some interplay between TLRs and adipokines. Lin et al. [42] suggested adipocyte *Tlr*2 synthesis increases upon stimulation with *Tnf*. Also, the expression of *Tlr*1-9 and *Tlr*11-13 in murine adipose tissue appears to be strongly regulated by leptin [43]. In the present study, the expression of *Tlr*1, *Tlr*2, *Tlr*6, *Tlr*7, *Tlr*8 and *Tlr*13 were significantly up-regulated by HFD in the epididymal WAT depot, along with *Tnf* and *Lep* expression, while expression of *Adipoq* was down-regulated. HFD also significantly up-regulated *Irf5* in the later stages of diet-induced obesity. *Irf5* is generally involved downstream of the TLR–MyD88 signalling pathway for induction of pro-inflammatory cytokines, and *Irf5* is a potential target of therapeutic interventions to control harmful immune responses [44].

Of the thirteen TLRs, TLR4 was characterized first, working in conjunction with the LPS receptor CD14, TLR4 is the primary signalling receptor for gram negative bacterial lipopolysaccharide (LPS) [45]. CD14 is crucial for LPS recognition by TLR4 and cooperates with other TLRs, including TLR2 and TLR3 [46,47]. Cd180 also belongs to the family of pathogen receptors. Cd180 takes part in B-cell recognition and LPS signalling [48]. In this study, we show diet-induced obesity leads to significant induction of *Cd14Cd180Cd68* and other Cd antigen genes active in the immune and inflammation system of animals. Therefore, expression of Cd antigen genes may be used as markers of visceral adipose tissue inflammation during the development of diet-induced obesity. Taken together these findings suggest that, adipose tissue expansion, and concomitant activation of TLR-mediated inflammatory signalling cascades and induction of CD antigens, causes increased expression of pro-inflammatory cytokines and chemokines, which results in chronic low-grade inflammation.

Obesity is in most, but not all humans, associated with marked changes in the secretory function of adipocytes and macrophages, together with chronic low-grade inflammation and an increased risk of developing fibrosis. A complex network of inflammatory cytokines, adipokines, transcription factors, and receptor molecules is involved in the development of adipose tissue fibrosis. In adipose tissue, fibrosis appears to be initiated in response to adipocyte hypertrophy. Adipose tissue ECM remodelling, which plays a pivotal role in adipogenesis and tissue architecture [49], is crucial to accommodate obesity-induced cellular alterations [50]. However, the persistence of an inflammatory stimulus in adipose tissue may be responsible for the excessive synthesis of ECM components and subsequent interstitial deposition of fibrotic material. Fibrosis, attributed to excessive deposition of ECM proteins, is a ubiquitous tissue response to chronic inflammation [51]. In this study, ECM gene expression was altered by HFD in a time-dependent manner. Furthermore, there was evidence of accumulation of fibrillar collagens, interspersed in between adipocytes in the visceral WAT of HFD-fed mice. Lumican (*Lum)* has a stimulatory effect on the epithelial-mesenchymal transition state of fibrosis, while decorin (*Dcn*) has an inhibitory role in transforming growth factor β (TGF-β)-induced fibrosis [52,53]. *Lum* mRNA levels were up-regulated, while *Dcn* mRNA levels were down-regulated at an early stage during the development of diet-induced obesity. *Tgfb1* mRNA levels were also up-regulated in the visceral WAT of HFD-fed mice. The up-regulation of *Lum* and *Tgfb1* mRNA levels and the down-regulation of *Dcn* mRNA levels were consistent with the observed development of fibrosis in visceral WAT of HFD-fed mice. Many metalloproteases (MMPs) were also altered at the mRNA level by HFD. *Mmp2, 3, Mmp12* and *Mmp13* are reportedly positively correlated with the degree of obesity and were up-regulated in the visceral WAT of HFD fed mice [54]. Interestingly, our detailed time-course microarray analysis over 24 weeks of HFD feeding showed that collagen mRNA levels were up-regulated shortly after the initiation of HFD feeding and then down-regulated by an unknown mechanism after 2 weeks. We suggest that up-regulation of collagen mRNA levels by HFD during the early stages of diet-induced obesity may trigger the accumulation of ECM components that ultimately leads to fibrosis which was evident in the later stages of diet-induced obesity.

The proteolytic and destructive properties of the lysosomal cathepsins play a role in degenerative, as well as chronic inflammatory diseases. Furthermore, proteases of the cathepsin family are involved in the remodelling of extracellular matrix (ECM) proteins [55]. This study indicates that members of the cathepsin family, including *CtsbCtskCtslCtss* and *Ctsz* as well as *Ctsa* may contribute to the pro-inflammatory and extracellular matrix remodelling in visceral WAT depots during the development of diet-induced obesity. Destruction of elastin-rich tissues during the inflammatory response is associated with local accumulation of macrophages that contain high levels of elastinolytic enzymes, such as Ctsb and Ctsl [56]. Ctss, known to degrade several components of the ECM, is also now well established as a novel biomarker of adiposity [57]. The function of cathepsin A in obesity is relatively unknown, however increases in expression of cathepsin B, D, K, S, and Z have previously been observed in obesity [58].

It is possible that some of differences in this study compared to others could be due to the lower fat content and different diet composition. Especially, while other studies used 60 kcal% fat diet consisting of soybean oil and lard, we used 39.2 kcal% from fat diet consisting of corn oil and lard. One example, several previous studies have found that hypoxia has been observed in obese adipose tissue and HIF1α (hypoxia-inducible factor 1α), a master signal mediator of hypoxia response, is significantly elevated in obese adipose tissue [4,59,60]. We also found that HIF1α gene expression tended to be higher (fold change 1.36 at HFD vs ND) at 24 week of high-fat feeding (about 39.2 kcal% from fat), but there was no statistically significant difference between the groups. However, in another experiment of ours that used commercial high fat diet with higher fat content and different fat composition (Research diets, Product No. D12451, 45 kcal% fat, 2.9% soybean oil and 20.7% lard), high fat feeding for 16 weeks significantly up-regulated HIF1α expression in adipose tissue of C57BL/6 J mice (Y. J. Kim, M. S. Choi, U. J. Jung, unpublished paper).

## Conclusions

The present findings indicate excessive fat accumulation occurs in visceral WAT depots (epidydimal, perirenal, retroperitoneum, mesentery) early in response to HFD feeding. Pro- and anti-inflammatory adipokines including leptin and adiponectin are differentially expressed in visceral WAT and are closely associated with plasma adipokine levels during the development of diet-induced obesity. However, much wider changes occur in the immune transcriptome of visceral WAT depots, which are early and sustained. Up-regulated inflammatory genes include pro-inflammatory cytokines, chemokines and their upstream signalling pathway genes. Early changes also occur in fibrosis-related genes, extracellular matrix (ECM), collagen and cathepsin genes, but fibrosis appears in the later stages of diet-induced obesity. New therapeutic approaches targeting visceral adipose tissue genes associated with inflammation and fibrosis which are altered early during adipose tissue accumulation may help ameliorate the deleterious effects of diet-induced obesity.

## Abbreviations

DEGs: Differentially expressed genes; ND: Normal diet; HFD: High-fat diet; WAT: White adipose tissue; ECM: Extracellular matrix.

## Competing interests

The author(s) declare that they have no competing interests.

## Authors' contributions

EYK performed experiments and wrote the manuscript. SKS and YYC carried out the animal experiments. UJJ, EK and RAM participated in writing the manuscript. TP, JHYP, JWY and YBP analyzed the data. EYK and MSC designed the study. All authors read and approved the final manuscript.

## Supplementary Material

Additional file 1 **Table S1.**Composition of experimental diets. ^1^AIN-76 mineral mixture (grams/kg): calcium phosphate 500, sodium chloride 74, potassium citrate 2220, potassium sulfate 52, magnesium oxide 24, magnesium carbonate 3.5, ferric citrate 6, zinc carbonate 1.6, cupric carbonate 0.3, potassium iodate 0.01, sodium celenite 0.01, chromium potassium sulfate 0.55, sucrose 118.03, ^2^AIN-76 vitamin mixture (grams/kg): thiamin HCL 0.6, riboflavin 0.6, pyridoxin HCL 0.7, niacin 3, calcium pantothenate 1.6, folic acid 0.2, biotin 0.02, vit B_12_ 1, vit A(500,000U/gm) 0.8, vit D_3_(400,000U/gm) 0.25, vit E acetate(500 U/gm) 10, menadione sodium bisulfite 0.08, sucrose 981.15, ND: normal diet (AIN-76), HFD: high-fat diet (20% fat, 1% cholesterol) Click here for file

Additional file 2 **Table S2.**Primer sequences used for RT-qPCR validation of microarray data. Click here for file

Additional file 3 **Table S3.**Body weight gain, food intake and food efficiency in C57BL/6 J mice fed the different diets for 24 weeks. Data shown as means ± S.D. Values are significantly different from the ND group according to Student's t-test: *p < 0.05, **p < 0.01, ***p < 0.001. ND: normal diet (AIN-76), HFD: high-fat diet (20% fat, 1% cholesterol), FER: food efficiency ratio. Click here for file

Additional file 4 **Table S4.**List of high-fat diet responding gene transcripts in epididymal WAT that simultaneously associated with advancing age in ND and HFD groups for 24 weeks. Click here for file

## References

[B1] Hotamisligil GS Inflammation and metabolic disorders Nature 2006 444 7121 860 867 10.1038/nature05485 17167474

[B2] Rosen ED Spiegelman BM Adipocytes as regulators of energy balance and glucose homeostasis Nature 2006 444 7121 847 853 10.1038/nature05483 17167472 PMC3212857

[B3] Maury E Brichard SM Adipokine dysregulation, adipose tissue inflammation and metabolic syndrome Mol Cell Endocrinol 2010 314 1 1 16 10.1016/j.mce.2009.07.031 19682539

[B4] Hosogai N Fukuhara A Oshima K Miyata Y Tanaka S Segawa K Furukawa S Tochino Y Komuro R Matsuda M Shimomura I Adipose tissue hypoxia in obesity and its impact on adipocytokine dysregulation Diabetes 2007 56 4 901 911 10.2337/db06-0911 17395738

[B5] Schönherr E Hausser HJ Extracellular matrix and cytokines: a functional unit Dev Immunol 2000 7 2–4 89 101 11097204 10.1155/2000/31748 PMC2276054

[B6] Okuda S Languino LR Ruoslahti E Border WA Elevated expression of transforming growth factor-beta and proteoglycan production in experimental glomerulonephritis. Possible role in expansion of the mesangial extracellular matrix J Clin Invest 1990 86 2 453 462 10.1172/JCI114731 2200803 PMC296747

[B7] Border WA Noble NA Transforming growth factor-beta in glomerular injury Exp Nephrol 1994 2 1 13 17 8081992

[B8] Tsochatzis EA Papatheodoridis GV Archimandritis AJ Adipokines in nonalcoholic steatohepatitis: from pathogenesis to implications in diagnosis and therapy Mediators Inflamm 2009 831670 1 9 Published online 10.1155/2009/831670 PMC2694309 19753129

[B9] Schram K Sweeney G Implications of myocardial matrix remodeling by adipokines in obesity-related heart failure Trends Cardiovasc Med 2008 18 6 199 205 10.1016/j.tcm.2008.10.001 19185809

[B10] Kleemann R van Erk M Verschuren L van den Hoek AM Koek M Wielinga PY Jie A Pellis L Bobeldijk-Pastorova I Kelder T Toet K Wopereis S Cnubben N Evelo C van Ommen B Kooistra T Time-resolved and tissue-specific systems analysis of the pathogenesis of insulin resistance PLoS One 2010 5 1 e8817 10.1371/journal.pone.0008817 20098690 PMC2809107

[B11] Khan T Muise ES Iyengar P Wang ZV Chandalia M Abate N Zhang BB Bonaldo P Chua S Scherer PE Metabolic dysregulation and adipose tissue fibrosis: role of collagen VI Mol Cell Biol 2009 29 6 1575 1591 10.1128/MCB.01300-08 19114551 PMC2648231

[B12] Wang B Wood IS Trayhurn P Dysregulation of the expression and secretion of inflammation-related adipokines by hypoxia in human adipocytes Pflugers Arch 2007 455 3 479 492 10.1007/s00424-007-0301-8 17609976 PMC2040175

[B13] Fitzgibbons TP Kogan S Aouadi M Hendricks GM Straubhaar J Czech MP Similarity of mouse perivascular and brown adipose tissues and their resistance to diet-induced inflammation Am J Physiol Heart Circ Physiol 2011 301 4 H1425 H1437 10.1152/ajpheart.00376.2011 21765057 PMC3197360

[B14] MacLaren RE Cui W Lu H Simard S Cianflone K Association of adipocyte genes with ASP expression: a microarray analysis of subcutaneous and omental adipose tissue in morbidly obese subjects BMC Med Genomics 2010 3 3 10.1186/1755-8794-3-3 20105310 PMC2843642

[B15] Halberg N Khan T Trujillo ME Wernstedt-Asterholm I Attie AD Sherwani S Wang ZV Landskroner-Eiger S Dineen S Magalang UJ Brekken RA Scherer PE Hypoxia-inducible factor 1alpha induces fibrosis and insulin resistance in white adipose tissue Mol Cell Biol 2009 29 16 4467 4483 10.1128/MCB.00192-09 19546236 PMC2725728

[B16] Do GM Kwon EY Kim E Kim HS Choi MS Hepatic transcription response to high-fat treatment in mice: microarray comparison of individual vs. pooled RNA samples Biotechnol J 2010 5 970 973 10.1002/biot.201000046 20715078

[B17] Gentleman R, Carey V, Huber W, Irizarry R, Dudoit S Bioinformatics and Computational Biology Solutions using R and Bioconductor 2005 Springer, New York

[B18] Schmittgen TD Livak KJ Analyzing real-time PCR data by the comparative C(T) method Nat Protoc 2008 3 1101 1108 10.1038/nprot.2008.73 18546601

[B19] Huang DW Sherman BT Lempicki RA Systematic and integrative analysis of large gene lists using DAVID bioinformatics resources Nat Protoc 2009 4 44 57 19131956 10.1038/nprot.2008.211

[B20] Do GM Oh HY Kwon EY Cho YY Shin SK Park HJ Jeon SM Kim E Hur CG Park TS Sung MK McGregor RA Choi MS Long-term adaptation of global transcription and metabolism in the liver of high-fat diet-fed C57BL/6 J mice Mol Nutr Food Res 2011 55 Suppl 2 S173 S185 21618427 10.1002/mnfr.201100064

[B21] Divoux A Clément K Architecture and the extracellular matrix: the still unappreciated components of the adipose tissue Obes Rev 2011 12 5 e494 e503 10.1111/j.1467-789X.2010.00811.x 21366833

[B22] Zhang F Basinski MB Beals JM Briggs SL Churgay LM Clawson DK DiMarchi RD Furman TC Hale JE Hsiung HM Schoner BE Smith DP Zhang XY Wery JP Schevitz RW Crystal structure of the obese protein leptin-E100 Nature 1997 387 6629 206 209 10.1038/387206a0 9144295

[B23] Tartaglia LA Dembski M Weng X Deng N Culpepper J Devos R Richards GJ Campfield LA Clark FT Deeds J Muir C Sanker S Moriarty A Moore KJ Smutko JS Mays GG Wool EA Monroe CA Tepper RI Identification and expression cloning of a leptin receptor, OB-R Cell 1995 83 7 1263 1271 10.1016/0092-8674(95)90151-5 8548812

[B24] Zarkesh-Esfahani H Pockley G Metcalfe RA Bidlingmaier M Wu Z Ajami A Weetman AP Strasburger CJ Ross RJ High-dose leptin activates human leukocytes via receptor expression on monocytes J Immunol 2001 167 8 4593 4599 11591788 10.4049/jimmunol.167.8.4593

[B25] Loffreda S Yang SQ Lin HZ Karp CL Brengman ML Wang DJ Klein AS Bulkley GB Bao C Noble PW Lane MD Diehl AM Leptin regulates proinflammatory immune responses FASEB J 1998 12 1 57 65 9438411

[B26] Oka T Nishimura Y Zang L Hirano M Shimada Y Wang Z Umemoto N Kuroyanagi J Nishimura N Tanaka T Diet-induced obesity in zebrafish shares common pathophysiological pathways with mammalian obesity BMC Physiol 2010 21 10 21 20961460 10.1186/1472-6793-10-21 PMC2972245

[B27] Steppan CM Bailey ST Bhat S Brown EJ Banerjee RR Wright CM Patel HR Ahima RS Lazar MA The hormone resistin links obesity to diabetes Nature 2001 409 6818 307 312 10.1038/35053000 11201732

[B28] Steppan CM Lazar MA The current biology of resistin J Intern Med 2004 255 4 439 447 10.1111/j.1365-2796.2004.01306.x 15049878

[B29] Jackson MB Osei SY Ahima RS The endocrine role of adipose tissue: focus on adiponectin and resistin Curr Opin Endocrin Diab 2005 12 163 170 10.1097/01.med.0000159113.87201.e8

[B30] Kawanami D Maemura K Takeda N Harada T Nojiri T Imai Y Manabe I Utsunomiya K Nagai R Direct reciprocal effects of resistin and adiponectin on vascular endothelial cells: a new insight into adipocytokine-endothelial cell interactions Biochem Biophys Res Commun 2004 314 2 415 419 10.1016/j.bbrc.2003.12.104 14733921

[B31] Kadowaki S Li SH Wang CH Fedak PW Li RK Weisel RD Mickle DA Resistin promotes endothelial cell activation: further evidence of adipokine-endothelial interaction Circulation 2003 108 6 736 740 10.1161/01.CIR.0000084503.91330.49 12874180

[B32] Kadowaki T Yamauchi T Kubota N Hara K Ueki K Tobe K Adiponectin and adiponectin receptors in insulin resistance, diabetes, and the metabolic syndrome J Clin Invest 2006 116 7 1784 1792 10.1172/JCI29126 16823476 PMC1483172

[B33] Bolduc C Yoshioka M St-Amand J Acute molecular mechanisms responsive to feeding and meal constitution in mesenteric adipose tissue Obesity (Silver Spring) 2010 18 2 410 413 10.1038/oby.2009.257 20111028

[B34] Sumiyoshi M Sakanaka M Kimura Y Chronic intake of high-fat and high-sucrose diets differentially affects glucose intolerance in mice J Nutr 2006 136 3 582 587 16484528 10.1093/jn/136.3.582

[B35] Park SY Cho YR Kim HJ Higashimori T Danton C Lee MK Dey A Rothermel B Kim YB Kalinowski A Russell KS Kim JK Unraveling the temporal pattern of diet-induced insulin resistance in individual organs and cardiac dysfunction in C57BL/6 mice Diabetes 2005 54 12 3530 3540 10.2337/diabetes.54.12.3530 16306372

[B36] Flier JS Cook KS Usher P Spiegelman BM Severely impaired adipsin expression in genetic and acquired obesity Science 1987 237 4813 405 8 10.1126/science.3299706 3299706

[B37] Maslowska M Vu H Phelis S Sniderman AD Rhode BM Blank D Cianflone K Plasma acylation stimulating protein, adipsin and lipids in non-obese and obese populations Eur J Clin Invest 1999 29 8 679 686 10.1046/j.1365-2362.1999.00514.x 10457151

[B38] Napolitano A Lowell BB Damm D Leibel RL Ravussin E Jimerson DC Lesem MD Van Dyke DC Daly PA Chatis P Concentrations of adipsin in blood and rates of adipsin secretion by adipose tissue in humans with normal, elevated and diminished adipose tissue mass Int J Obes Relat Metab Disord 1994 18 4 213 218 8044195

[B39] Cianflone K Xia Z Chen LY Critical review of acylation-stimulating protein physiology in humans and rodents Biochim Biophys Acta 2003 1609 2 127 143 10.1016/S0005-2736(02)00686-7 12543373

[B40] Caesar R Manieri M Kelder T Boekschoten M Evelo C Müller M Kooistra T Cinti S Kleemann R Drevon CA A combined transcriptomics and lipidomics analysis of subcutaneous, epididymal and mesenteric adipose tissue reveals marked functional differences PLoS One 2010 5 7 e11525 10.1371/journal.pone.0011525 20634946 PMC2902507

[B41] Morton NM Nelson YB Michailidou Z Di Rollo EM Ramage L Hadoke PW Seckl JR Bunger L Horvat S Kenyon CJ Dunbar DR A stratified transcriptomics analysis of polygenic fat and lean mouse adipose tissues identifies novel candidate obesity genes PLoS One 2011 6 9 e23944 10.1371/journal.pone.0023944 21915269 PMC3168488

[B42] Lin Y Lee H Berg AH Lisanti MP Shapiro L Scherer PE The lipopolysaccharide-activated toll-like receptor (TLR)-4 induces synthesis of the closely related receptor TLR-2 in adipocytes J Biol Chem 2000 275 32 24255 24263 10.1074/jbc.M002137200 10823826

[B43] Batra A Pietsch J Fedke I Glauben R Okur B Stroh T Zeitz M Siegmund B Leptin-dependent toll-like receptor expression and responsiveness in preadipocytes and adipocytes Am J Pathol 2007 170 6 1931 1941 10.2353/ajpath.2007.060699 17525261 PMC1899459

[B44] Takaoka A Yanai H Kondo S Duncan G Negishi H Mizutani T Kano S Honda K Ohba Y Mak TW Taniguchi T Integral role of IRF-5 in the gene induction programme activated by Toll-like receptors Nature 2005 434 7030 243 249 10.1038/nature03308 15665823

[B45] Beutler B Tlr4: central component of the sole mammalian LPS sensor Curr Opin Immunol 2000 12 1 20 26 10.1016/S0952-7915(99)00046-1 10679411

[B46] Miyake K Roles for accessory molecules in microbial recognition by Toll-like receptors J Endotoxin Res 2006 12 4 195 204 10.1179/096805106X118807 16953972

[B47] Akashi-Takamura S Miyake K TLR accessory molecules Curr Opin Immunol 2008 20 4 420 425 10.1016/j.coi.2008.07.001 18625310

[B48] Ogata H Su I Miyake K Nagai Y Akashi S Mecklenbräuker I Rajewsky K Kimoto M Tarakhovsky A The toll-like receptor protein RP105 regulates lipopolysaccharide signaling in B cells J Exp Med 2000 192 1 23 29 10.1084/jem.192.1.23 10880523 PMC1887709

[B49] Pierleoni C Verdenelli F Castellucci M Cinti S Fibronectins and basal lamina molecules expression in human subcutaneous white adipose tissue Eur J Histochem 1998 42 3 183 188 9857243

[B50] Lijnen HR Angiogenesis and obesity Cardiovasc Res 2008 78 2 286 293 10.1093/cvr/cvm007 18006485

[B51] Wynn TA Common and unique mechanisms regulate fibrosis in various fibroproliferative diseases J Clin Invest 2007 117 3 524 529 10.1172/JCI31487 17332879 PMC1804380

[B52] Kolb M Margetts PJ Sime PJ Gauldie J Proteoglycans decorin and biglycan differentially modulate TGF-beta-mediated fibrotic responses in the lung Am J Physiol Lung Cell Mol Physiol 2001 280 6 L1327 L1334 11350814 10.1152/ajplung.2001.280.6.L1327

[B53] Rada JA Cornuet PK Hassell JR Regulation of corneal collagen fibrillogenesis in vitro by corneal proteoglycan (lumican and decorin) core proteins Exp Eye Res 1993 56 6 635 648 10.1006/exer.1993.1081 8595806

[B54] Chavey C Mari B Monthouel MN Bonnafous S Anglard P Van Obberghen E Tartare-Deckert S Matrix metalloproteinases are differentially expressed in adipose tissue during obesity and modulate adipocyte differentiation J Biol Chem 2003 278 14 11888 11896 Epub 10.1074/jbc.M209196200 12529376

[B55] McGrath ME The lysosomal cysteine proteases Annu Rev Biophys Biomol Struct 1999 28 181 204 10.1146/annurev.biophys.28.1.181 10410800

[B56] Fiebiger E Maehr R Villadangos J Weber E Erickson A Bikoff E Ploegh HL Lennon-Duménil AM Invariant chain controls the activity of extracellular cathepsin L J Exp Med 2002 196 9 1263 1269 10.1084/jem.20020762 12417635 PMC2194106

[B57] Taleb S Lacasa D Bastard JP Poitou C Cancello R Pelloux V Viguerie N Benis A Zucker JD Bouillot JL Coussieu C Basdevant A Langin D Clement K Cathepsin S, a novel biomarker of adiposity: relevance to atherogenesis FASEB J 2005 19 11 1540 1542 15985526 10.1096/fj.05-3673fje

[B58] Nadler ST Stoehr JP Schueler KL Tanimoto G Yandell BS Attie AD The expression of adipogenic genes is decreased in obesity and diabetes mellitus Proc Natl Acad Sci USA 2000 97 21 11371 11376 10.1073/pnas.97.21.11371 11027337 PMC17207

[B59] Rausch ME Weisberg S Vardhana P Tortoriello DV Obesity in C57BL/6 J mice is characterized by adipose tissue hypoxia and cytotoxic T-cell infiltration Int J Obes (Lond) 2008 32 3 451 463 10.1038/sj.ijo.0803744 17895881

[B60] Ye J Gao Z Yin J He Q Hypoxia is a potential risk factor for chronic inflammation and adiponectin reduction in adipose tissue of ob/ob and dietary obese mice Am J Physiol Endocrinol Metab 2007 293 4 E1118 E1128 10.1152/ajpendo.00435.2007 17666485

