# Supplementary material for: Time-course microarrays reveal early activation of the immune transcriptome and adipokine dysregulation leads to fibrosis in visceral adipose depots during diet-induced obesity
Source: BMC Genomics. 2012 Sep 4;13:450. doi: 10.1186/1471-2164-13-450 (PMC3447724; doi:10.1186/1471-2164-13-450)
Supplement: Additional file 1 — Table S1.Composition of experimental diets. 1AIN-76 mineral mixture (grams/kg): calcium phosphate 500, sodium chloride 74, potassium citrate 2220, potassium sulfate 52, magnesium oxide 24, magnesium carbonate 3.5, ferric citrate 6, zinc carbonate 1.6, cupric carbonate 0.3, potassium iodate 0.01, sodium celenite 0.01, chromium potassium sulfate 0.55, sucrose 118.03, 2AIN-76 vitamin mixture (grams/kg): thiamin HCL 0.6, riboflavin 0.6, pyridoxin HCL 0.7, niacin 3, calcium pantothenate 1.6, folic acid 0.2, biotin 0.02, vit B12 1, vit A(500,000U/gm) 0.8, vit D3(400,000U/gm) 0.25, vit E acetate(500 U/gm) 10, menadione sodium bisulfite 0.08, sucrose 981.15, ND: normal diet (AIN-76), HFD: high-fat diet (20% fat, 1% cholesterol) [file 1471-2164-13-450-S1.pdf]

Table S1 Composition of experimental diets

Ingredient (g)	ND	HFD
Casein	200.00	200.00
D,L-methionine	3.00	3.00
Corn starch	150.00	111.00
Sucrose	500.00	370.00
Cellulose powder	50.00	50.00
Corn oil	50.00	30.00
Lard	—	170.00
Mineral mixture (AIN-76) [1]	35.00	42.00
Vitamin mix (AIN-76) [2]	10.00	12.00
Choline bitartrate	2.00	2.00
Cholesterol	—	10.00
tert-Butylhydroquinone	0.01	0.04
Total (g)	1,000.0	1,000.0

[1]AIN-76 mineral mixture (grams/kg); calcium phosphate 500, sodium chloride 74, potassium citrate 2220, potassium sulfate 52, magnesium oxide 24, magnesium carbonate 3.5, ferric citrate 6, zinc carbonate 1.6, cupric carbonate 0.3, potassium iodate 0.01, sodium celenite 0.01, chromium potassium sulfate 0.55, sucrose 118.03

[2]AIN-76 vitamin mixture (grams/kg); thiamin HCL 0.6, riboflavin 0.6, pyridoxin HCL 0.7, niacin 3, calcium pantothenate 1.6, folic acid 0.2, biotin 0.02, vit B_{12} 1, vit A(500,000U/gm) 0.8, vit D_3(400,000U/gm) 0.25, vit E acetate(500 U/gm) 10, menadione sodium bisulfite 0.08, sucrose 981.15

ND; normal diet (AIN-76), HFD; high-fat diet (20% fat, 1% cholesterol)
