# Supplementary material for: Time-course microarrays reveal early activation of the immune transcriptome and adipokine dysregulation leads to fibrosis in visceral adipose depots during diet-induced obesity
Source: BMC Genomics. 2012 Sep 4;13:450. doi: 10.1186/1471-2164-13-450 (PMC3447724; doi:10.1186/1471-2164-13-450)
Supplement: Additional file 2 — Table S2.Primer sequences used for RT-qPCR validation of microarray data. [file 1471-2164-13-450-S2.pdf]

Table S2 Primer sequences used for RT-qPCR validation of microarray data

Gene	Primer direction	Primer sequence
Glyceraldehyde-3-phosphate dehydrogenase (*Gapdh*)	Forward	5'-ACAATGAAT ACGGCTACAGCAACAG-3'
	Reverse	5'-GGTGGTCCAGGGTTTCTTACTCC-3'
Leptin (*Lep*)	Forward	5'-AGGGAGGAAAATGTGCTGGAGAC-3'
	Reverse	5'-GATACCGACTGCGTGTGTGAAATG-3'
Adiponectin (*Adipoq*)	Forward	5'-GGTCTTCTTGGTCCTAAGGGTGAG-3'
	Reverse	5'-GCGGCTTCTCCAGGCTCTC-3'
Resistin (*Retn*)	Forward	5'-GCTGCTCCTGTGGCTCTGC-3'
	Reverse	5'-GGCTGCTGTCCAGTCTATCCTTG-3'
