# Supplementary material for: Time-course microarrays reveal early activation of the immune transcriptome and adipokine dysregulation leads to fibrosis in visceral adipose depots during diet-induced obesity
Source: BMC Genomics. 2012 Sep 4;13:450. doi: 10.1186/1471-2164-13-450 (PMC3447724; doi:10.1186/1471-2164-13-450)
Supplement: Additional file 3 — Table S3.Body weight gain, food intake and food efficiency in C57BL/6 J mice fed the different diets for 24 weeks. Data shown as means ± S.D. Values are significantly different from the ND group according to Student's t-test: *p < 0.05, **p < 0.01, ***p < 0.001. ND: normal diet (AIN-76), HFD: high-fat diet (20% fat, 1% cholesterol), FER: food efficiency ratio. [file 1471-2164-13-450-S3.pdf]

Table S3 Body weight gain, food intake and food efficiency in C57BL/6J mice fed the different diets for 24 weeks.

		0wk	2wks	4wks	6wks	8wks	12wks	16wks	20wks	24wks
Body weight (g)	ND	18.02±0.39	20.86±0.22	24.3-±0.31	25.63±0.26	27.60±0.55	31.32±0.61	32.48±0.89	35.29±0.99	33.43±0.86
	HFD		21.45±0.32	25.52±0.44*	27.14±0.60*	30.39±0.78**	36.28±0.80***	42.44±1.71**	44.43±1.35***	45.92±0.60***
Food intake (g)	ND	-	4.09±0.13	3.83±0.17	3.75±0.05	3.91±0.08	3.84±0.08	3.77±0.10	3.79±0.07	4.15±0.07
	HFD		3.29±0.12***	3.05±0.09***	3.21±0.06***	2.99±0.09***	3.00±0.06***	3.17±0.14**	3.27±0.10***	3.16±0.09***
FER	ND	-	0.09±0.003	0.06±0.003	0.05±0.003	0.05±0.002	0.04±0.001	0.03±0.002	0.03±0.001	0.02±0.002
	HFD		0.10±0.006	0.09±0.006***	0.07±0.004***	0.08±0.003***	0.97±0.002***	0.06±0.004***	0.06±0.002***	0.06±0.002***

Data shown as means ± S.D. Values are significantly different from the ND group according to Student's test: *p<0.05, **p<0.01, ***p<0.001.

ND; normal diet (AIN-76), HFD; high-fat diet (20% fat, 1% cholesterol), FER; food efficiency ratio.
